# The Interaction Between Nitrogen Supply and Light Quality Modulates Plant Growth and Resource Allocation

**DOI:** 10.3389/fpls.2022.864090

**Published:** 2022-05-04

**Authors:** Ying Liang, C. Mariano Cossani, Victor O. Sadras, Qichang Yang, Zheng Wang

**Affiliations:** ^1^Institute of Urban Agriculture, Chinese Academy of Agriculture Sciences, Chengdu, China; ^2^Vegetable Germplasm Innovation and Variety Improvement Key Laboratory of Sichuan, Horticulture Research Institute, Sichuan Academy of Agricultural Sciences, Chengdu, China; ^3^South Australian Research and Development Institute, and School of Agriculture, Food and Wine, The University of Adelaide, Adelaide, SA, Australia

**Keywords:** blue light, red light, biomass allocation, nitrogen allocation, nitrogen concentration, lettuce

## Abstract

Nitrogen availability and light quality affect plant resource allocation, but their interaction is poorly understood. Herein, we analyzed the growth and allocation of dry matter and nitrogen using lettuce (*Lactuca sativa* L.) as a plant model in a factorial experiment combining three light regimes (100% red light, R; 50% red light + 50% blue light, RB; 100% blue light, B) and two nitrogen rates (low, 0.1 mM N; high, 10 mM N). Red light increased shoot dry weight in relation to both B and RB irrespective of nitrogen supply. Blue light favored root growth under low nitrogen. Allometric analysis showed lower allocation to leaf in response to blue light under low nitrogen and similar leaf allocation under high nitrogen. A difference in allometric slopes between low nitrogen and high nitrogen in treatments with blue light reflected a strong interaction effect on root-to-shoot biomass allocation. Shoot nitrate concentration increased with light exposure up to 14 h in both nitrogen treatments, was higher under blue light with high nitrogen, and varied little with light quality under low nitrogen. Shoot nitrogen concentration, nitrogen nutrition index, and shoot NR activity increased in response to blue light. We conclude that the interaction between blue light and nitrogen supply modulates dry mass and nitrogen allocation between the shoot and root.

## Introduction

Control environment agriculture allows for whole-year production and high yield, factors that are particularly important for valuable horticultural crops (Kozai et al., 2019). Nitrogen supply and light (intensity and spectral composition) are important in these intensive production systems (Tsukagoshi and Shinohara, 2020). The individual effects of nitrogen and light quality on plant growth, nitrogen uptake, and allocation of both dry matter and reduced nitrogen attract large research efforts (Hogewoning et al., 2010; Wang et al., 2016; Zhou et al., 2018; Bian et al., 2020). However, less attention has been paid to the interactions between nitrogen supply and light quality on plant growth and resource allocation.

Biomass partitioning is central to plant fitness and adaptation (Korner, 1991, 2015), and increased allocation to grain has been at the core of the improvement of yield in grain crops over the last decades (Liu et al., 2021; Slafer et al., 2021). The relative allocation to shoot and root influences the capture of below-ground and above-ground resources (Titlyanova et al., 1999; Husáková et al., 2018), and reflects the cumulative response of plant growth to variable environments (Mokany et al., 2006). Partitioning between structural and labile carbohydrates is ecologically and agronomically relevant, with reserves playing a role in re-growth after fire or herbivory, grain fill, and osmotic defense against aphids (Bloom et al., 1985; Sadras, 2021). Sugar signaling, interacting with nutrients and light, modulates the partitioning of carbon between functions, including growth, defense, and reserves (Smith and Stitt, 2007).

Plants absorb radiation efficiently through chlorophylls in the 600–700 nm (red) 400–500 nm (blue) wavelengths (McCree, 1971), and an array of photoreceptors perceive different wavebands of sunlight with roles in photomorphogenesis. Phytochromes mediate perception of red and far-red light, while cryptochromes (CRY), phototropins, and members of the ZTL/FKF1/LKP2 family mediate perception of UVA and blue light (Rai et al., 2019; Hernando et al., 2021; Pierik and Ballare, 2021). Monochromatic blue light, red light, or their mixture influence photosynthesis, plant morphology, and dry mass allocation between organs and between structural and labile carbohydrates, but their effects seem inconsistent (Hogewoning et al., 2010; Wang et al., 2016; He et al., 2017). Izzo et al. (2020) reported that red light reduced tomato root growth and root-to-shoot ratio compared to the mixture of red light with blue light. Pham et al. (2019) found blue light reduced root weight compared to red light and mixed red and blue light in tomato seedlings. Sakuraba and Yanagisawa (2018) reviewed the effects of light signaling on sucrose transportation via expression of sugar transporter by HY5, which could induce phloem-mobile sucrose in leaf to promote root development and nitrate uptake (Lejay et al., 2003; Chen et al., 2012; Kircher and Schopfer, 2012).

Nitrogen deficit often increases the root-to-shoot ratio (Poorter et al., 2012; Sun et al., 2020). Increasing evidence indicated that nitrate and light coordinately regulate plant growth. Light signals can induce genes involved in nitrate metabolism and influence nitrogen uptake and assimilation (Sakuraba and Yanagisawa, 2018; Pathak et al., 2020). Both red and blue light showed a positive effect on nitrate metabolism (Kamiya, 1989; Jonassen et al., 2008). Yuan et al. (2016) reported that blue light regulated flowering in plants in response to nitrogen levels. The effect of the interaction between nitrogen and light on horticultural crops is poorly understood. In this study, we used lettuce (*Lactuca Sativa* L.) as a model plant to quantify the growth, nitrogen uptake, and shoot–root allocation of dry matter and nitrogen in a factorial experiment combining light quality and nitrogen supply, with a particular focus on interactions.

## Materials and Methods

Two experiments were conducted in a growth room with a controlled environment to test the effect of nitrogen supply, light quality, and their interaction on lettuce growth, nitrogen uptake, and allocation of both dry matter and reduced nitrogen for a short term (treatment for 1 week, Exp. 1) and long term (treatment for 3 weeks, Exp. 2). Both experiments had the same design.

### Growing Conditions and Experimental Design

**Figure 1:**
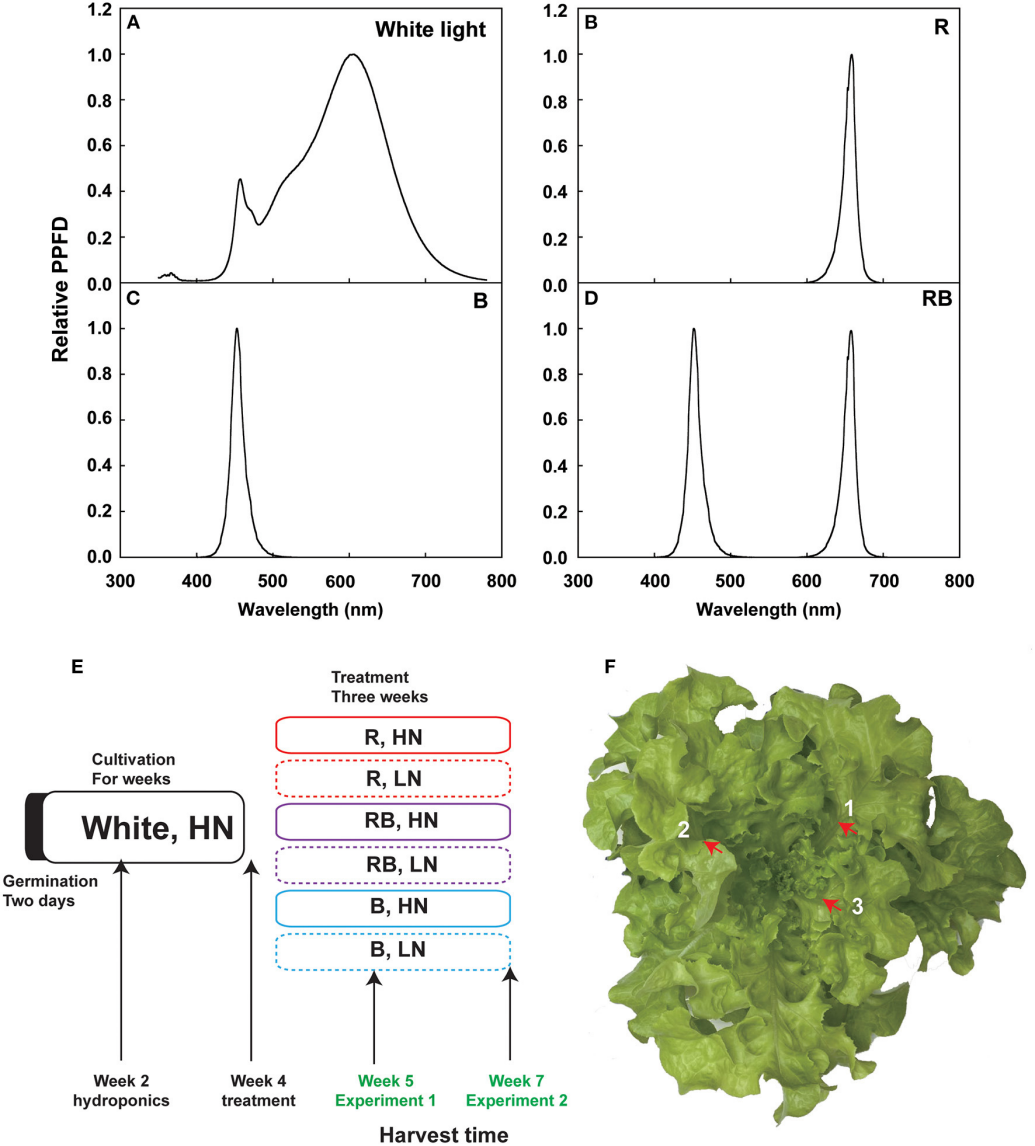
Overview of the experiment. **(A–D)** Spectra of the LED lights used in this experiment, including white light, R, RB, and B. **(E)** Experimental schedule of light and nitrogen treatments in Experiments 1 and 2. Germinated lettuce seeds were sowed in the sponge grown in the white LED for 2 weeks, irrigated with HN nutrition, and then transferred the plants to nutrient solution for another 2 weeks. Four-week-old lettuce plants were treated with different light qualities and nitrogen concentrations for 1 week (Exp. 1) and 3 weeks (Exp. 2) before harvest. **(F)** Three newest expanded leaves are labeled with a number. Red arrow shows the main vein, which was removed.

Lettuce (*Lactuca Sativa* L. cv. “Green Oak leaf”) seeds were sowed in a 0.25 cm × 0.25 cm × 0.25 cm sponge (Jiangsu Rongcheng Agricultural Science and Technology Development Co. Ltd, Nantong, Jiangsu, P. R. China) and germinated in darkness for 2 days in a growth room with day/night temperatures of 24/22°C, CO_2_ concentration of 400 ppm, and vapor pressure deficit (VPD) of 1.19/1.06 kPa. Upon unfolding of the first true leaf (2 weeks after sowing), treatments were established and maintained for 1 week (Exp. 1) or 3 weeks (Exp. 2) (Figure 1E). Plants were watered with a nutrient solution for 2 weeks under photosynthetic photon flux density (PPFD) of 200 μmol m^−2^ s^−1^ white LED light (light spectrum in Figure 1A) and a photoperiod of 14 h, 24/20°C day/night temperature, and VPD 1.19/0.94 kPa before transferring to a hydroponic system with the same conditions. The nutrient solution (10 mM nitrate-N) was renewed weekly and contained 4 mM KNO_3_, 0.8 mM KH_2_PO_4_, 0.3 mM K_2_HPO_4_, 1.5 mM MgSO_4_, 3 mM Ca (NO_3_)_2_, 0.08 mM Fe-Na EDTA, 60 μM H_3_BO_3_, 3 μM ZnSO_4_, 20 μM MnSO_4_, 0.4 μM CuSO_4_, and 0.03 μM (NH_4_)_6_Mo_7_O_24_, with pH 5.8 and EC 1.38 mS·cm^−1^.

After 2 weeks of cultivation, vigorous and uniform plants were selected for the light and nitrogen treatments. Three cultivation frames were used, each divided into three layers, and two tanks were placed in each layer (18 cultivation units in total). Light conditions were 100% red light (R), 50% red light and 50% blue light (RB), and 100% blue light (B) (light spectrum in Figures 1B–D). Lighting equipment was red and blue LED light tubes (150 cm × 60 cm, Shenzhen CT Lighting Technology Co. Ltd., Shenzhen, Guangdong, P. R. China), with peak wavelengths of 659 nm and 450 nm, respectively. Light quality and intensity were measured using a spectroradiometer (SS120, Apogee Instruments, Inc., Logan, UT, USA). Light intensity at the canopy level was maintained at 200 μmol m^−2^ s^−1^. To avoid light contamination, each cultivation unit was covered with an opaque black–white plastic film. Nitrogen conditions were low nitrogen (LN, 0.1 mM nitrate-N) and high nitrogen (HN, 10 mM nitrate-N). Low nitrogen treatments were established by replacing KNO_3_ with KCl and Ca(NO_3_)_2_ with CaCl_2_ to maintain the same K^+^ and Ca^2+^ concentrations. Plastic tanks with 15 L of plant nutrient solution were used for the nitrogen treatment. Six plants were planted in each tank. An automatic pump was used to maintain the oxygen content in the nutrient solution.

### Sampling and Harvesting

Plants from one tank were harvested as one sample, and three samples were used during the experiment. In Exp. 1, after 8 days of treatment, (Figure 1E), shoot and root samples were collected to determine the concentration of nitrate and soluble sugar at 0, 6, and 14 h after the onset of the light period. Nitrate reductase (NR; 1.6.6.1) activity was measured in the 6-h sample. For the sampling of shoots, the newest three expanded leaves were collected, and the leaf veins were removed with scissors (Figure 1F). Roots were collected and blotted dry with tissue to remove the excess nutrient solution. Fresh samples were snap-frozen in liquid nitrogen for the enzymatic and chemical analyses.

For the destructive measurements, plants were harvested on the 8th day (Exp. 1) and the 21st day (Exp. 2) after the treatment (Figure 1E). Plants were divided into shoot and root in Exp.1. The shoot was divided into three parts in Exp. 2: old, expanded, and unexpanded leaves. Old leaves were the first five leaves and unexpanded leaves were the newest unexpanded 3–4 leaves, while the remaining leaves were the expanded leaves. We measured fresh weight and dry weight after drying on a forced air oven at 65°C until a constant weight was obtained. The dried samples were milled with a TISSUELYSER-24/32L (Shanghai Jingxin Industrial Development Co. Ltd., Shanghai, China) at 60 Hz for 1 min for chemical analysis.

### Chemical Analysis

We measured leaf and root nitrate concentration with the salicylic acid method (Cataldo et al., 1975). Frozen leaf/root samples (0.2 g) were homogenized using a TISSUELYSER-24/32L (Shanghai Jingxin Industrial Development Co. Ltd., Shanghai, China) at 60 Hz for 1 min and incubated with 1 ml of water at 25°C for 30 min. Then the samples were centrifuged at 10,000 × *g* for 15 min. The reaction mixture contains 10 μl of supernatant, 40 μl of reaction buffer, and 950 μl of H_2_SO_4_. The absorbance of the extract was measured at 410 nm using a UV–VIS spectrophotometer (UV-1800, Shimadzu, Japan). Nitrogen concentration was measured in 0.1 g dried samples digested with concentrated sulfuric acid (98%) and hydrogen peroxide (≥30%) using the micro-Kjeldahl procedure. Soluble sugar content was analyzed with the anthrone method (Ruuska et al., 2006). The homogenized frozen leaf/root samples (0.2 g) were incubated in 1.5 ml of 80% ethanol in a 50°C water bath for 20 min, and then centrifuged at 10,000 × *g* for 10 min. The supernatants were used to determine the soluble sugar content at the absorbance of 620 nm.

### NR Activity

In a preliminary trial, we noticed that the NR activity decreased significantly after storage, especially at −80°C. To minimize this effect, the activity of NR was tested immediately after harvest. The NR activity was determined using the commercial reaction agent (www.geruisi-bio.com) according to the manufacturer's instructions. Briefly, 0.1 g of sample was homogenized in 1 ml of extraction buffer (pH 7.5, containing 100 mM Hepes-KOH, 7 mM cysteine, 1 mM EDTA, 3% PVPP (polyvinylpolypyrrolidone), and 1% BSA) and centrifuged at 10,000 × *g* for 10 min. Then, 80 μl of the supernatant was mixed with 400 μl of reaction buffer containing potassium nitrate and NADH. Another tube with 80 μl of the supernatant mixed with 400 μl of reaction buffer and containing only potassium nitrate without NADH was used as the control. All the tubes were incubated at 30°C for 30 min in the dark. The content of ${\text{NO}}_{2}^{-}$ was determined at the absorbance of 530 nm by adding 400 μl of color agents (1% sulfanilamide and 0.02% N-1-naphthyl-ethylene-diamine dihydrochloride in 1.5 M HCl). The difference in the ${\text{NO}}_{2}^{-}$ value between the assay tubes and control tubes indicates the nitrate content that is reduced by the extracted enzyme solution. The amount of ${\text{NO}}_{2}^{-}$ produced by the enzyme solution per minute indicates the NR enzyme activity.

### Data Analysis

Following the definition of Poorter and Sack (2012), we calculated leaf mass fraction (LMF) as the ratio of shoot dry weight to total dry weight, and root mass fraction (RMF) as the ratio of root dry weight to total dry weight. The N nutrition index (NNI) was calculated as the ratio of actual and critical N concentration in the shoot at 1 week and 3 weeks. Critical N content was derived from the N dilution curve for lettuce reported by Yin et al. (2021). Shapiro–Wilk test was performed to verify normality (when *p* > 0.05, normal distribution), and the Levene's test (when *p* > 0.05, equal variance) was conducted to verify the homogeneity of variances. If *p* < 0.05, the data were log-transformed to meet the equal variance assumption. Two-way ANOVA was used to evaluate the effect of nitrogen supply, light quality, and their interaction on plant biomass, allocation-related traits, and nitrogen-related traits. One-way ANOVA was used to evaluate the effect of light quality and diurnal variation on nitrate concentration and soluble sugar concentration in shoot and root. Principal component analysis was performed with GraphPad Prism 9 to explore the relationships among morphological, physiological, and nitrogen traits and their responses to treatments. Correlations between traits were explored with Model II linear regression to account for errors in both x- and y-axes (Ludbrook, 2012).

## Results

### Effect of Nitrogen Supply and Light Quality on Plant Biomass Accumulation and Allocation

**Figure 2:**
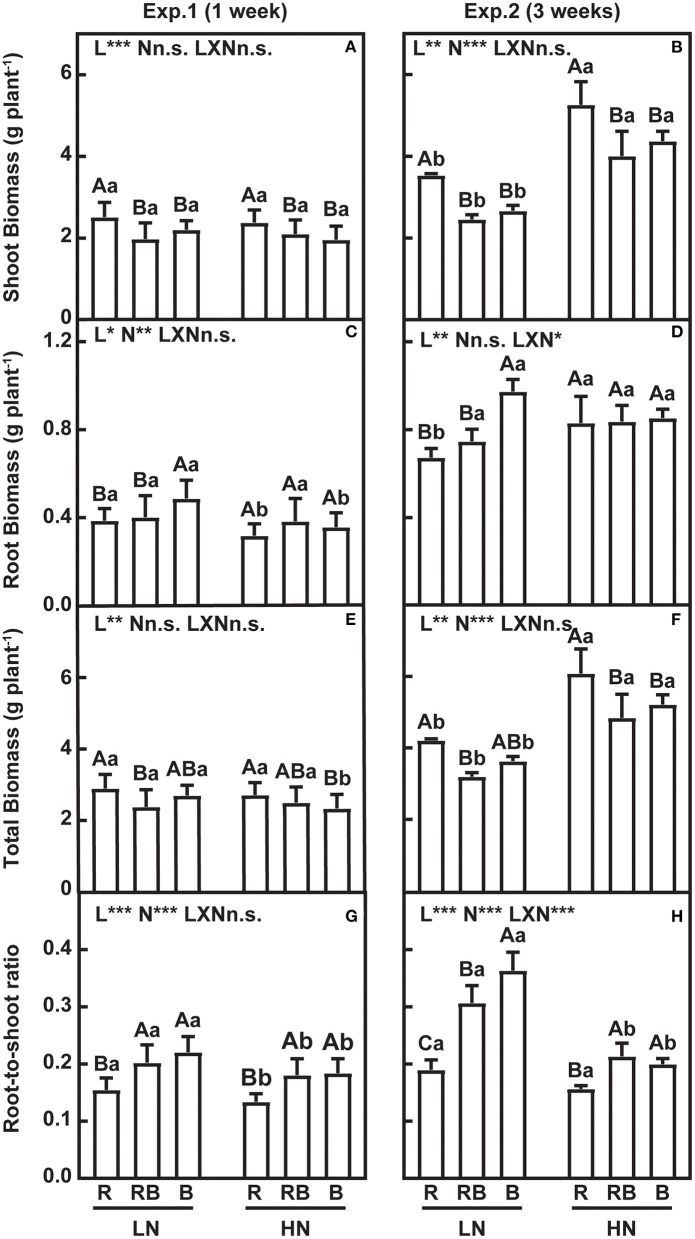
Growth and root-to-shoot ratio of lettuce in a factorial experiment combining three light and two nitrogen treatments in Experiments 1 and 2. **(A–F)** Shoot, root, and total dry weight (DW). **(G,H)** Root-to-shoot ratio. Error bars indicate one standard deviation (*n* = 12 and 3 in Exp. 1 and Exp. 2, respectively). Lower case letters are used for comparison between nitrogen input under the same light quality, and upper case letters for comparison among light treatments under the same nitrogen input. Different letters indicate differences at *p* = 0.05 according to Fischer's LSD test. Effects of light, nitrogen, and interaction from ANOVA: ***p < 0.001, **p < 0.01, *p ≤ 0.05, and n.s. *p* > 0.05.

The effects of nitrogen, light quality, and their interaction are summarized in Figure 2. Shoot dry weight (DW) was higher in R compared to RB and B after 1 week and 3 weeks of treatment, irrespective of the nitrogen supply (Figures 2A,B). Under low nitrogen, root DW was higher with blue light after 1 week and 3 weeks of treatment (Figures 2C,D). Under high nitrogen, the light quality did not affect the root DW (Figures 2C,D). Differences in total DW were incipient after 1 week, and after 3 weeks, total DW was higher under red light irrespective of nitrogen and higher under high nitrogen irrespective of light (Figures 2E,F). After 1 week, the root-to-shoot ratio (R/S) was lowest under red light and high nitrogen, with no interaction (Figure 2G). After 3 weeks, an interaction was apparent whereby R/S had a coefficient of variation of 0.28 and ranked R < RB < B under low nitrogen, in comparison to the coefficient of variation of 0.15 and ranking R < RB ≈ B under high nitrogen.

**Figure 3:**
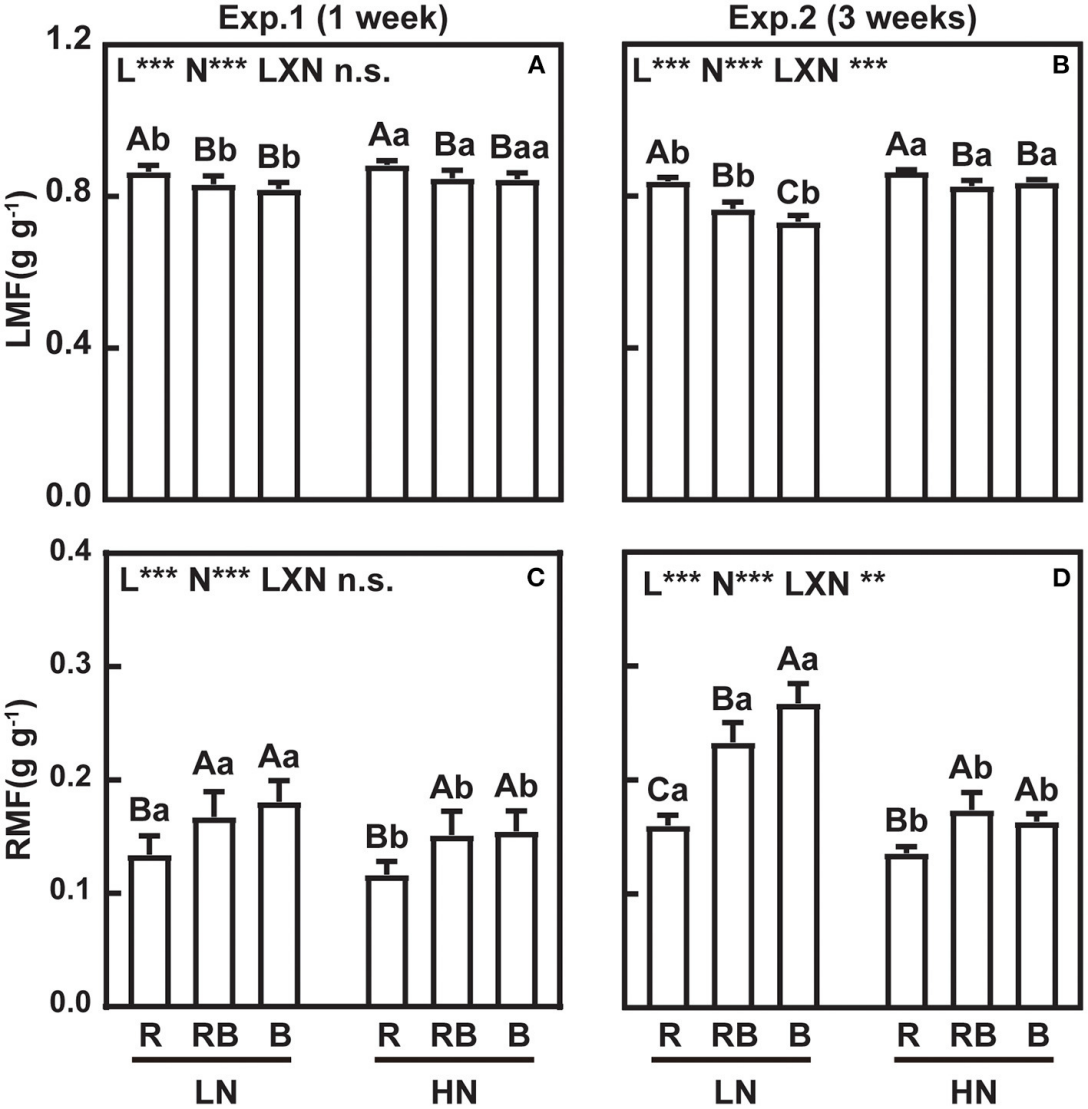
LMF (leaf mass fraction) and RMF (root mass fraction) of lettuce in a factorial experiment combining three light and two nitrogen treatments in Experiments 1 and 2. **(A,B)** LMF and **(C,D)** RMF. Error bars indicate one standard deviation (*n* = 12 and 3 in Exp. 1 and Exp. 2, respectively). Lower case letters are used for comparison between nitrogen input under the same light quality, and upper case letters for comparison among light treatments under the same nitrogen input. Different letters indicate differences at *p* = 0.05 according to Fischer's LSD test. Effects of light, nitrogen, and interaction from ANOVA: ***p < 0.001, **p < 0.01, *p ≤ 0.05, and n.s. *p* > 0.05.

**Figure 4:**
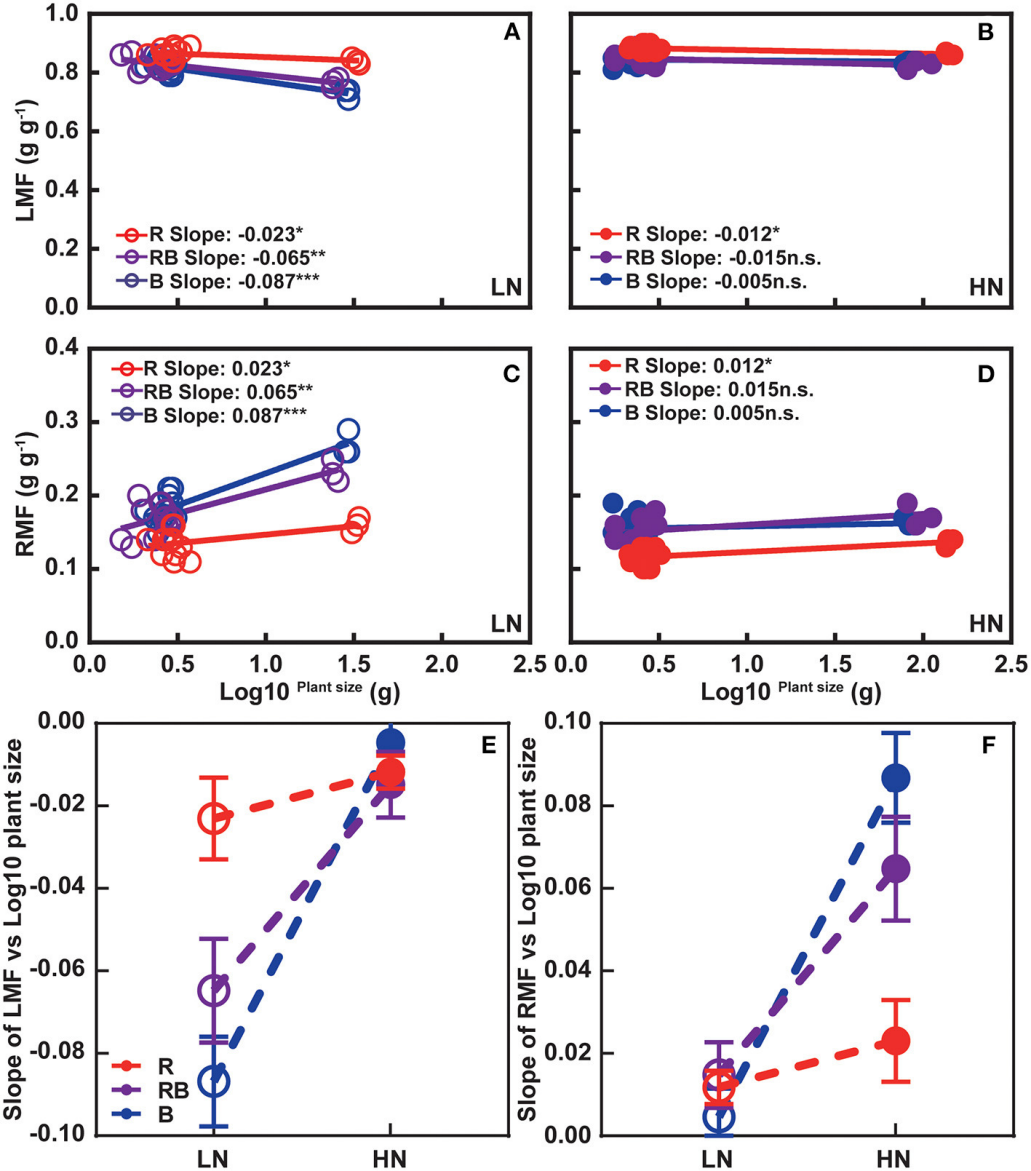
Allometric relations between **(A,B)** LMF and **(C,D)** RMF with plant size for plants grown under **(A,C)** high and **(B,D)** low nitrogen. **(E,F)** Comparison of allometric slopes in response to light quality and nitrogen supply. In **(A–D)**, data are pooled across 1-week and 3-week experiments. Open and solid circles indicate LN and HN, respectively. Lines are least square regressions. Significance of slope is ***p < 0.001, **p < 0.01, *p ≤ 0.05, and n.s. *p* > 0.05. In **(E,F)** open and solid circles indicate allometric slope in LN and HN treatments, respectively, and error bars are one standard deviation of the allometric slope.

Figure 3 shows leaf mass fraction (LMF) and root mass fraction (RMF) in response to treatments. LMF was highest and RMF lowest under red light after 1 week irrespective of the nitrogen treatment. After 3 weeks, an interaction developed whereby LMF ranked R > RB > B at low nitrogen and R > RB ≈ B under high nitrogen, with a mirror response for RMF. Figure 4 further analyzes allocation to leaf and root to account for size-dependent effects (Poorter and Sack, 2012). Under low nitrogen, the slope of LMF against the log of plant weight ranked RB ≈ B < R, whereas the slopes were similar under high nitrogen (Figures 4A,B). The slopes of RMF against the log of plant weight were the mirror images of those for LMF. Next, we compared the allometric slopes for R, RB, and B under high and low nitrogen (Figures 4E,F). The allometric slope of LMF did not vary with light treatment under high nitrogen, but the slope was higher under red light compared to RB and B under low nitrogen.

### Nitrogen Concentration, Nitrogen Content, and Nitrogen Allocation in Response to Nitrogen Supply and Light Quality

**Figure 5:**
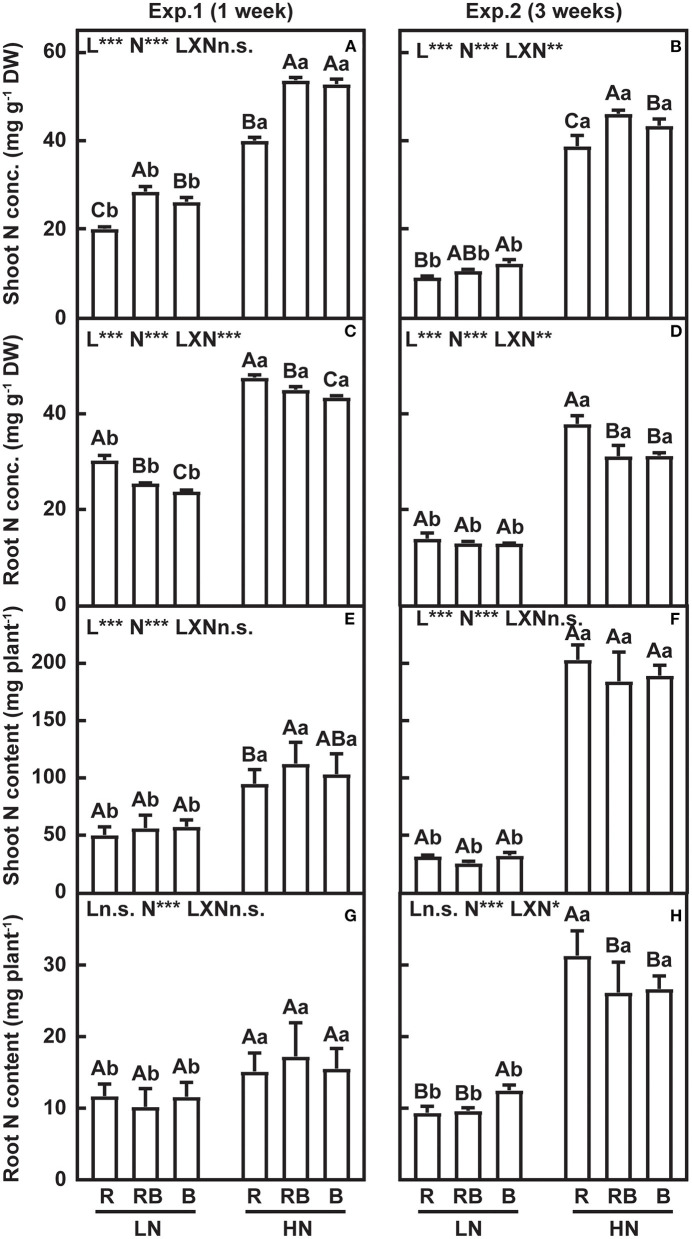
Nitrogen concentration and nitrogen content of lettuce in a factorial experiment combining three light and two nitrogen treatments in Experiments 1 and 2. **(A,B)** Shoot and **(C,D)** root nitrogen concentration. **(E,F)** Shoot and **(G,H)** root nitrogen content. Error bars indicate one standard deviation (*n* = 3). Lower case letters are used for comparison between nitrogen input under the same light quality, and upper case letters for comparison among light treatments under the same nitrogen input. Different letters indicate differences at *p* = 0.05 according to Fischer's LSD test. Effects of light, nitrogen, and interaction from ANOVA: ***p < 0.001, **p < 0.01, *p ≤ 0.05, and n.s. *p* > 0.05.

The effect of nitrogen, light quality, and their interaction on nitrogen traits are summarized in Figure 5. Shoot nitrogen concentration was lowest and root nitrogen concentration was highest in R compared to RB and B after 1 week and 3 weeks of treatment, except for lack of variation in root nitrogen concentration with low nitrogen after 3 weeks of treatment (Figures 5A–D). After 1 week of treatment, shoot nitrogen content was lowest in R under high nitrogen, with no interactions. No interaction effect was detected in the shoot nitrogen content after 3 weeks of treatment (Figures 5E,F). After 1 week of treatment, root nitrogen content did not vary with light treatments (Figure 5G). After 3 weeks, an interaction developed whereby root nitrogen content ranked R < RB ≈ B at low nitrogen and R > RB ≈ B under high nitrogen (Figure 5H).

Nitrogen concentration in the old leaves was higher in RB than in R and B under both nitrogen supplies (Table 1). The nitrogen concentration of the expanded leaves and young leaves was lower in R compared to B under both nitrogen treatments. Nitrogen content of old leaves under low nitrogen was higher in R compared to B, while no differences in the nitrogen content were found for expanded and unexpanded leaves. Under high nitrogen, nitrogen content in RB was higher than in R and B in the old leaves; nitrogen content of B was higher in the expanded leaves and lower in the unexpanded leaves compared to R and RB.

**Table 1:** Nitrogen concentration and total nitrogen content in old, expanded, and unexpanded leaves in response to nitrogen input and light quality after 3 weeks of treatment.

**Trait**	**Nitrogen**	**Light**	**Old leaf**	**Expanded leaf**	**Unexpanded leaf**
Nitrogen concentration (% DW)	LN	R	0.90 ± 0.04b	0.71 ± 0.01c	1.13 ± 0.05b
		RB	1.13 ± 0.07a	0.85 ± 0.01b	1.25 ± 0.05b
		B	0.96 ± 0.04b	1.04 ± 0.09a	1.43 ± 0.05a
	HN	R	2.38 ± 0.11b	3.77 ± 0.17b	4.20 ± 0.19b
		RB	3.64 ± 0.49a	4.74 ± 0.02a	4.67 ± 0.09a
		B	2.65 ± 0.21b	4.47 ± 0.16a	4.48 ± 0.13ab
	Light		^**^	^***^	^**^
Effect	Nitrogen		^***^	^***^	^***^
	L × N		n.s.	^**^	^*^
Nitrogen content (mg plant-1)	LN	R	3.32 ± 0.48a	12.54 ± 0.21a	15.97 ± 1.43ab
		RB	3.06 ± 0.26ab	10.15 ± 0.55b	12.51 ± 0.80b
		B	2.43 ± 0.07b	12.27 ± 0.77a	17.63 ± 1.95a
	HN	R	7.82 ± 2.44b	99.75 ± 7.37b	95.56 ± 4.74a
		RB	15.85 ± 1.12a	98.72 ± 7.27b	69.84 ± 1.60b
		B	8.67 ± 1.98b	123.62 ± 5.84a	57.20 ± 0.97c
	Light		^*^	n.s.	^***^
Effect	Nitrogen		^***^	^***^	^***^
	L × N		^*^	n.s.	^**^

R is 100% red light, RB is 50% red light and 50% blue light, and B is 100% blue light. Mean ± standard deviation (n = 3); different letters indicate differences at p = 0.05 according to Fischer's LSD test. Effects of light, nitrogen, and interaction from ANOVA: ^***^p < 0.001, ^**^p < 0.01, ^*^ p ≤ 0.05, and n.s. p > 0.05.

*Old leaves were the first five leaves, unexpanded leaves were the newest unexpanded 3–4 leaves in the stem apex, and the remaining 5–7 (LN) and 9–11 (HN) leaves were the expanded leaves*.

### Effect of Nitrogen Supply and Light Quality on Nitrogen Nutrition Status and Partitioning of Nitrogen

**Figure 6:**
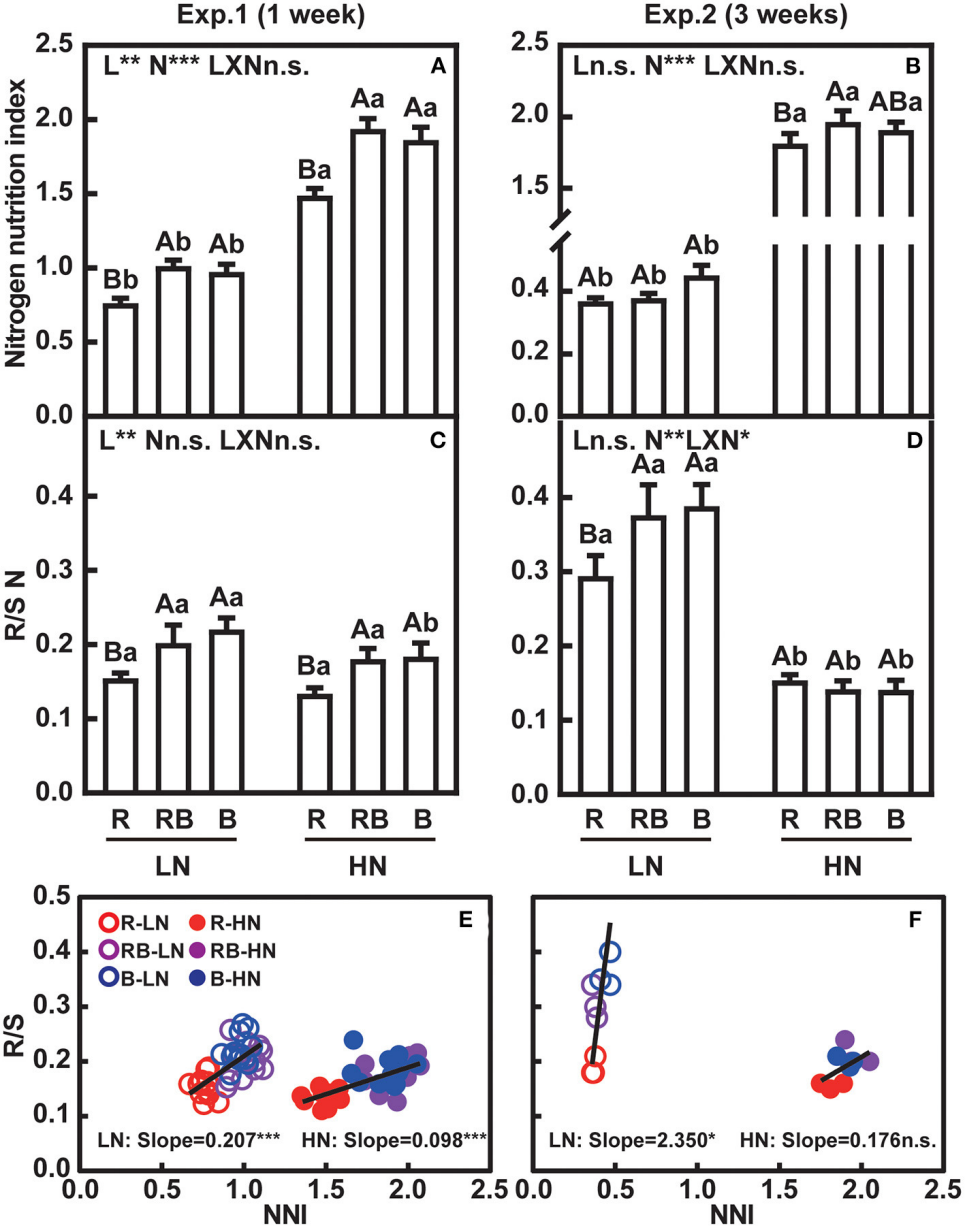
Nitrogen nutrition index and ratio of root-to-shoot nitrogen content of lettuce in a factorial experiment combining three light and two nitrogen treatments in Experiments 1 and 2. **(A,B)** Nitrogen nutrition index (NNI). **(C,D)** Root-to-shoot nitrogen content (R/S N). **(E,F)** Associations between root-to-shoot ratio and NNI. In **(A–D)**, error bars indicate one standard deviation (*n* = 12 and 3 in Exp. 1 and Exp. 2, respectively). Lower case letters are used for comparison between nitrogen input under the same light quality, and upper case letters for comparison among light treatments under the same nitrogen input. Different letters indicate differences at *p* = 0.05 according to Fischer's LSD test. Effects of light, nitrogen, and interaction from ANOVA: *** *p* < 0.001, ** *p* < 0.01, * *p* ≤ 0.05, and n.s. *p* > 0.05. In **(E,F)**, open and solid circles indicate LN and HN treatments, respectively. Lines are least square regressions, and significance is indicated as ***p < 0.001, **p < 0.01, *p ≤ 0.05, and n.s. *p* > 0.05.

The nitrogen nutrition index (NNI) accounts for the allometry between the concentration of nitrogen and dry matter, and is an unequivocal indicator of plant nitrogen status (Sadras and Lemaire, 2014). An NNI = 1 indicates that the plant nitrogen status is sufficient to meet maximum growth, whereas NNI < 1 indicates a deficiency, and NNI > 1 indicates excess nitrogen. After 1 week, plants with low nitrogen supply were close to or just below nitrogen sufficiency under low nitrogen, and they were nitrogen deficient after 3 weeks (NNI about 0.4) (Figures 6A,B). The high nitrogen treatment ensured no limitation (NNI > 1) at 1 and 3 weeks. After 1 week, red light reduced NNI irrespective of the nitrogen supply (Figure 6A). After 3 weeks, the effects of light and light × nitrogen interaction were not apparent for NNI (Figure 6B).

Figures 6C,D shows the partitioning of nitrogen between root and shoot. Root-to-shoot nitrogen content was lowest with red light irrespective of nitrogen after 1 week. After 3 weeks, an interaction was apparent whereby root-to-shoot nitrogen content remained lowest under low nitrogen, but light effects were no longer apparent under high nitrogen.

Linear correlation analysis was performed between NNI and R/S to evaluate resource allocation in response to nitrogen status. After 1 week, NNI and R/S correlated with a slope of 0.207 ± 0.037 under low nitrogen and 0.098 ± 0.014 under high nitrogen (Figure 6E). After 3 weeks, R/S correlated with NNI only under low nitrogen, with a slope of 2.35 ± 1.02 (Figure 6F).

### Effect of Nitrogen and Light Quality on Diurnal Changes of Nitrate Concentration, Soluble Sugar Concentration, and NR Activity

**Figure 7:**
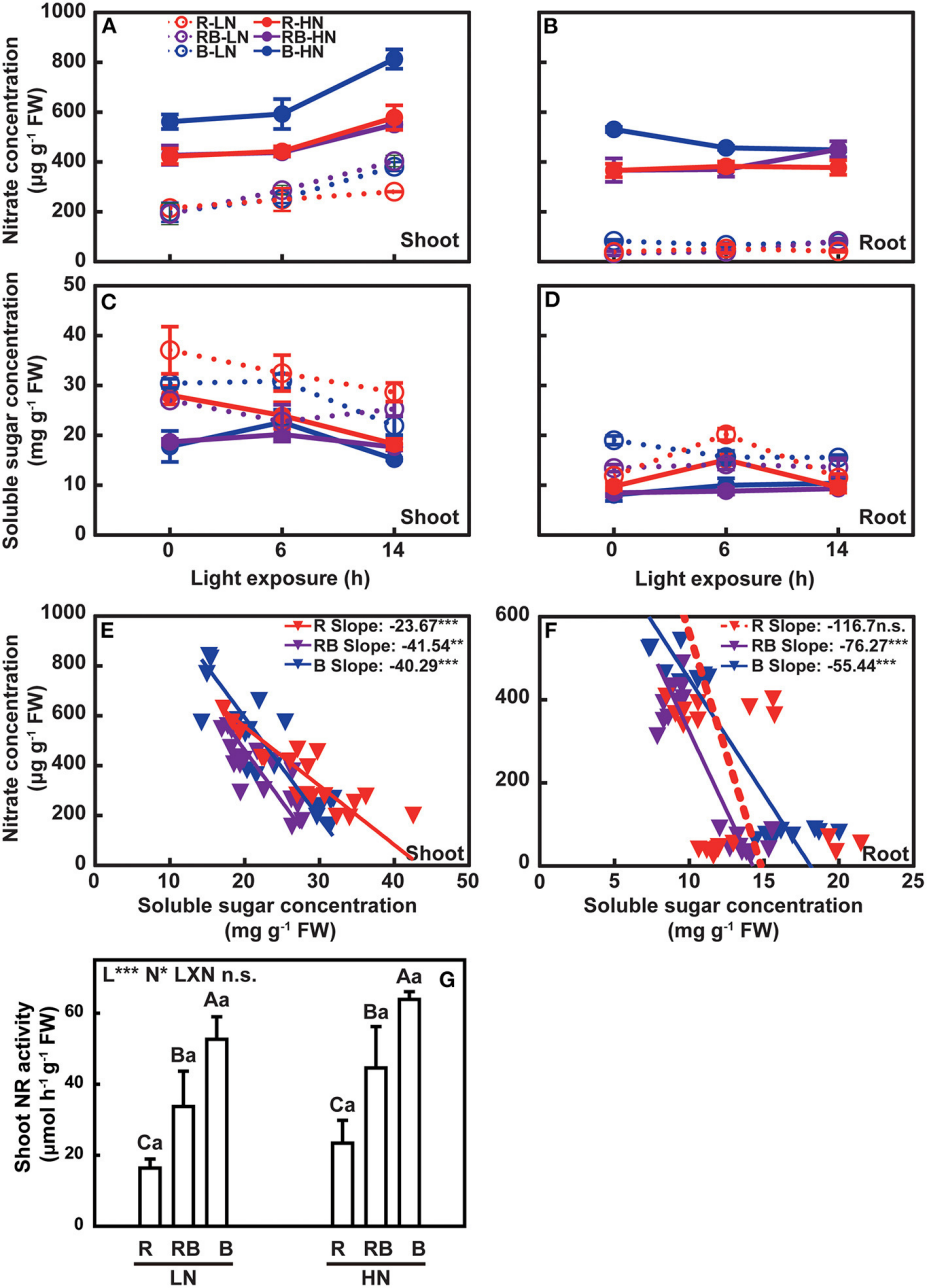
Nitrate concentration, soluble sugar concentration, and NR activity of lettuce in a factorial experiment combining three light and two nitrogen treatments in Exp. 1. Change in nitrate concentration in **(A)** shoot and **(B)** root with light exposure. Change in soluble sugar concentration in **(C)** shoot and **(D)** root with light exposure. Associations between nitrate and soluble sugar in **(E)** shoot and **(F)** root under three light treatments. **(G)** NR activity in the shoot. In **(A–D)**, open and solid circles indicate LN and HN treatments, respectively. Circles and lines with different colors indicate different light treatments. Error bars are one standard deviation (*n* = 3). In **(E,F)**, inverted triangles and lines with different colors indicate different light treatments. Lines are least square regressions with a slope not different from zero (dashed) or slopes different from zero (solid) (*p* = 0.05). In **(G)**, error bars indicate one standard deviation. Lower case letters are used for comparison between nitrogen input under the same light quality, and upper case letters for comparison among light treatments under the same nitrogen input. Different letters indicate differences at *p* = 0.05 according to Fischer's LSD test. Effects of light, nitrogen, and interaction from ANOVA: ***p < 0.001, **p < 0.01, *p ≤ 0.05, and n.s. *p* > 0.05.

In Exp. 1, the nitrate concentration of the newest three fully expanded leaves and root was determined at 0, 6, and 14 h after exposure to light treatments (Figures 7A,B); (Supplementary Tables S1,S2). Shoot nitrate concentration increased with light exposure in both nitrogen treatments, was higher under blue light in plants with high nitrogen, and varied little with light treatment under low nitrogen. Nitrate concentration was lower in root than in shoot, particularly under low nitrogen, with no apparent response to light treatment.

The concentration of sugar in shoot and root was higher in nitrogen-deficient plants and decreased with the duration of light exposure under low nitrogen (Figures 7C,D); (Supplementary Tables S1,S2). Soluble sugars in the root were less responsive to light exposure time (Figure 7D). Concentrations of sugars and nitrate were negatively correlated (Figures 7E,F). The nitrate-to-soluble sugar ratio of the red light deviated from that of blue light in a gentle slope (Figures 7E,F). Shoot NR activity increased with nitrogen supply, ranked B > RB > R, and did not vary with the interaction between light and nitrogen (Figure 7G).

### Associations Between Traits

**Figure 8:**
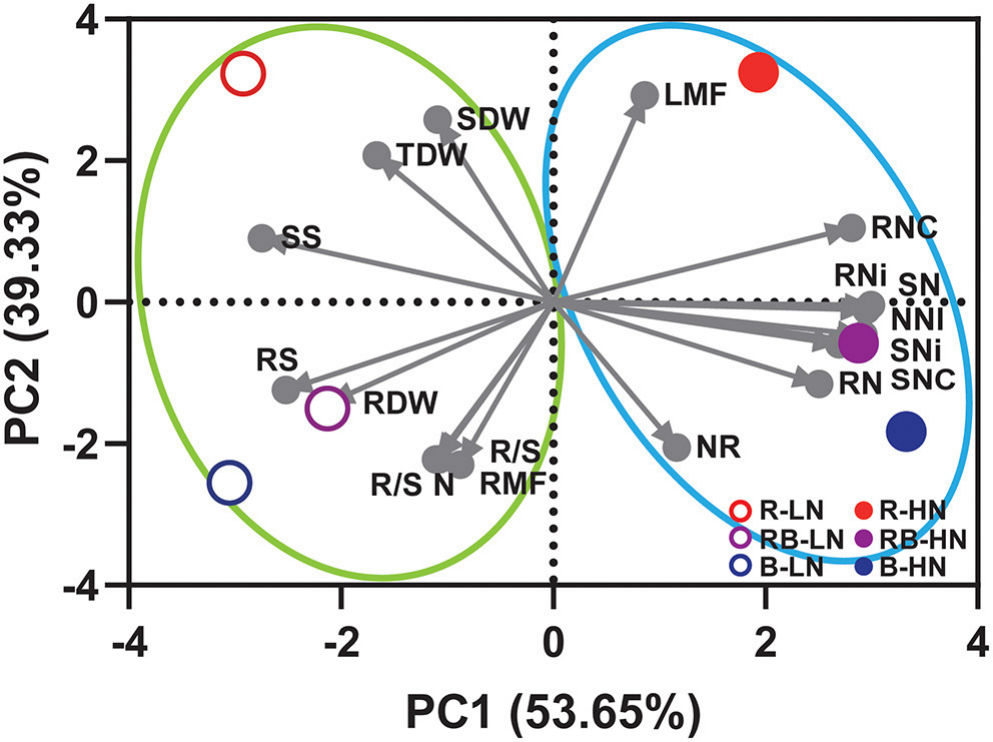
Principal component analysis of growth, nitrogen, and allocation-related traits of lettuce in response to light quality and nitrogen supply. SDW, shoot dry weight; RDW, root dry weight; TDW, total dry weight; R/S, root-to-shoot ratio; LMF, leaf mass fraction; RMF, root mass fraction; SNC, shoot nitrogen concentration; RNC, root nitrogen concentration; SN, shoot nitrogen content; RN, root nitrogen content; SNi, shoot nitrate concentration; RNi, root nitrate concentration; NNI, nitrogen nutrition index; R/S N, root-to-shoot nitrogen content; NR, shoot nitrate reductase activity; SS, shoot soluble sugar concentration; RS, root soluble sugar concentration.

Principal component analysis revealed clusters separated by nitrogen supply (PC1) and light quality (PC2) (Figure 8). The first principal component accounted for 53.65% of the total variance and the second for 39.33%. Low nitrogen supply, PC1(–), increased the root growth and soluble sugar concentration. High nitrogen supply, PC1(+), increased the nitrogen-related traits, including SNC, RNC, SN, RN, SNi, RNi, SNR, and NNI. Red light and blue light (RB and B) were separated along PC2. PC2(–) and PC2(+) contributed to the traits associated with the root (R/S, RMF) and shoot (SDW, LMF), respectively, suggesting blue light and red light modulate resource allocation differentially. Blue light [RB and B; PC2(–)] contributed to nitrogen-related traits, except RNC that is associated with red light.

## Discussion

Blue light and red light play different roles in resource allocation within shoot and root. In our study, red light increased shoot DW and reduced root DW compared to B and RB under both nitrogen supplies (Figure 2). Similar results were reported in the seedlings of lettuce (Chen et al., 2014) and other horticultural plants (Di et al., 2020; Gil et al., 2020; Izzo et al., 2020). Higher leaf area can over-compensate the reductions in photosynthesis with red light resulting in increased biomass (Hogewoning et al., 2010; Trouwborst et al., 2016; Wang et al., 2016). Monochromic blue light enhanced the root growth, compared to a monochromic red light or mixed light treatment (Izzo et al., 2020), which led to a higher root dry weight and root-to-shoot ratio in our blue light treatments (Figures 2C,G). Light quality modulates plant biomass allocation via photoreceptors; for example, in *Arabidopsis*, red light photoreceptor (phytochrome) regulated shoot growth, while blue light photoreceptor (cryptochrome) mediated the root elongation (Canamero et al., 2006; Yang et al., 2016). Nitrogen availability affects biomass allocation between root and shoot. Limited nitrogen supply inhibits shoot growth, while stimulating root elongation and lateral root formation, thus increasing the root-to-shoot ratio (Zhang and Forde, 2000; Bouguyon et al., 2016). In our study, root DW increased under low compared to high nitrogen in the 1-week treatment (Figure 2C), whereas shoot DW did not vary with nitrogen after 1 week of treatment, consistent with other studies (Zhou et al., 2018). In our study, which was conducted in 4-week-old lettuce plants, the nitrogen pool in leaves was sufficient to maintain the growth for a short period.

Root DW and root-to-shoot ratio responded to the interaction between nitrogen and light, whereas shoot DW did not (Figure 2). A similar result was reported in rocket leaves (Signore et al., 2020). To account for size-dependent variation in allocation, we related leaf mass fraction (LMF) and root mass fraction (RMF) to the log of plant DW (Poorter and Sack, 2012). At the same plant size, RMF was higher in RB and B compared to R (Figures 3C,D). Similar results were found with tomato seedlings where the blue light increased the RMF, although the root-to-shoot ratio was 0.14 in red light and 0.31 under blue light in the experiment by Izzo et al. (2020), and 0.10 and 0.08 in the study of Pham et al. (2019). The slope of RMF vs. log-total DW was higher in RB and B treatments under low nitrogen (Figure 4C). Allometric analysis in our study revealed a clear shift of slope from high nitrogen to low nitrogen in RB and B treatments, indicating an interaction between nitrogen and blue light on biomass allocation (Figures 4E,F). A synergy effect was demonstrated whereby the blue light, combined with nitrogen limitation, could increase the root dry weight and root-to-shoot ratio in lettuce.

A higher nitrogen accumulation in plant shoot grown under monochromic blue light has been reported in several crop plants (Wang et al., 2016; Chen et al., 2021; Liang et al., 2021). Under R, shoot nitrogen concentration was lower than under B and RB, while the root nitrogen concentration was higher (Figures 5A–D). Similar results were found by Signore et al. (2020) in rocket leaves. An interaction effect between light quality and nitrogen tended to be observed in N concentration, rather than content (Figure 5), indicating that shifts in nitrogen allocation were partly caused by the dilution effect of biomass (Figure 2). Nitrogen uptake is coregulated by nitrogen supply and crop biomass accumulation., and the NNI captures the nitrogen-biomass allometry (Lemaire et al., 2008). Few studies have explored the relationship between light quality and NNI. Our study demonstrated a significant improvement in NNI in RB and B treatments under both low and high nitrogen supply, which unambiguously demonstrates better nitrogen nutrition with blue light (Figure 6A). Furthermore, a higher root-to-shoot ratio of nitrogen content was also detected under B (Figures 6C–F). Blue light thus plays a positive role to maintain the nitrogen allocation from root to shoot, particularly under limited nitrogen supply.

Diurnal variation of nitrate absorption depends on the light intensity and duration (Okuyama et al., 2015). Nitrate concentration increased upon exposure to light (Reed et al., 1983; Kamiya, 1997). Blue light contributed to the maintenance of nitrate concentration in shoot under limited nitrogen supply compared to red light (Figure 7A). Previous studies indicated that red light induced a higher NR activity (Lillo and Appenroth, 2001; Sakuraba and Yanagisawa, 2018); however, the activation of NR activity in *Arabidopsis* by low levels of red light in the presence of sucrose in the growth medium suggests that carbon plays a dominant role in the activation of NR in the absent of blue light (Jonassen et al., 2008). In a non-photosynthetic mutant of *Chlorella kessleri*, blue light enhanced the uptake of nitrate, ammonium, amino acids, and nitrogen metabolism (Kamiya, 1988, 1989, 1995, 1997; Kamiya and Saitoh, 2002), which agrees with our findings (Figure 7G). Therefore, red light and blue light play different roles in nitrogen metabolism. Moreover, NR activity is regulated by several environmental factors, and the post-translational regulation of NR activity depends on light quality, rather than nitrate supply in the leaves (Kaiser and Huber, 2001). Appenroth et al. (2000) excluded the function of red light signal on post-translational regulation of NR. However, more evidence was needed to prove that blue light induced the NR activity at the post-translation level. In our experiment, the variation of NR activity was three-fold with light treatments compared to 43% with nitrogen supply (Figure 7G), highlighting the major role of blue light in regulating the NR activity.

Light quality modulated soluble sugar concentration, achieving higher concentration under monochromic red light (Figure 7C), as reported by previous studies (Wang et al., 2016; Chen et al., 2021). This phenomenon could be associated with a restriction of photosynthate translocation out of leaves and feedback inhibition of photosynthesis (Hogewoning et al., 2010; Wang et al., 2016; Liang et al., 2021). In *Arabidopsis*, phytochrome promotes cell wall formation and protein synthesis (Yang et al., 2016). In our study, a negative association between the concentration of nitrate and soluble sugars (Figures 7E,F) reflects a generalized, albeit poorly understood relation in plants (Hoogmoed and Sadras, 2016). Monochromic red light increased plant soluble sugar concentration associated with lower nitrate concentration, indicating that blue light played a role in maintaining the carbon: nitrogen (C:N) ratio (Hogewoning et al., 2010). HY5 is induced by blue light and coordinates leaf photosynthesis and root nitrogen uptake that favors the C:N balance in plants (Chen et al., 2016; Liang et al., 2021), consistent with our assumption that blue light is essential to main the C:N ratio in plants.

In summary, our research highlights the effects of the interactions between nitrogen supply and light quality on dry matter and nitrogen accumulation and allocation between shoot and root. Resource allocation is central to plant adaptation and crop yield. This study has potential applications in the design of light/nitrogen regimes for controlled environment horticulture.

## Data Availability Statement

The original contributions presented in the study are included in the article/Supplementary Material, further inquiries can be directed to the corresponding author/s.

## Author Contributions

YL and ZW conceived and designed the research, collected the data, prepared the figures, and wrote the manuscript. CC and VS contributed to the presentation, statistical analysis, and interpretation of the data, and writing of the manuscript. ZW and QY managed the project. All authors read and approved the manuscript.

## Funding

This research was funded by the National Key Research and Development Program, Ministry of Science and Technology of China (No. 2020YFE0203600), Sichuan Science and Technology Program (No. 2021YJ0500), the Agricultural Science and Technology Innovation Program of Chinese Academy of Agricultural Sciences (No. ASTIP-CAAS, 34-IUA), and the Central Public-Interest Scientific Institution Basal Research Fund (No. Y2021XK04).

## Conflict of Interest

The authors declare that the research was conducted in the absence of any commercial or financial relationships that could be construed as a potential conflict of interest.

## Publisher's Note

All claims expressed in this article are solely those of the authors and do not necessarily represent those of their affiliated organizations, or those of the publisher, the editors and the reviewers. Any product that may be evaluated in this article, or claim that may be made by its manufacturer, is not guaranteed or endorsed by the publisher.
