# Supplementary material for: The Interaction Between Nitrogen Supply and Light Quality Modulates Plant Growth and Resource Allocation
Source: Front Plant Sci. 2022 May 4;13:864090. doi: 10.3389/fpls.2022.864090 (PMC9115566; doi:10.3389/fpls.2022.864090)
Supplement: Supplementary file 1 [file Table_1.docx]

Table S1 **Concentration of nitrate and soluble sugars in lettuce shoot in response of duration of light exposure, nitrogen input and light quality.** Means ± standard deviation; different letters indicate differences at p = 0.05 according to Fischer’s l.s.d. with lower-case letters for comparison among exposure hour under same light quality, and capital letters for comparison among light treatments under same nitrogen input.

| Trait | Nitrogen | Light | Light exposure (h) | | |
| --- | --- | --- | --- | --- | --- |
|  |  |  | 0 | 6 | 14 |
| Nitrate concentration  (μg g^-1^ FW) | LN | R | 215±26.0 Aa | 249±37.6 Aa | 281±1.61 Ba |
|  |  | RB | 192±35.6 Ac | 287±15.6 Ab | 404±18.9 Aa |
|  |  | B | 199±31.7 Ac | 253±14.4 Ab | 382±14.8 Aa |
|  | HN | R | 423±25.0 Bb | 443±16.2 Bb | 579±40.1 Ba |
|  |  | RB | 427±31.8 Bb | 439±13.5 Bb | 550±7.20 Ba |
|  |  | B | 562±24.0 Ab | 593±49.2 Ab | 813±31.7 Aa |
| Soluble sugar concentration  (mg g^-1^ FW) | LN | R | 37.1±3.87 Aa | 32.5±2.96 Ab | 28.7±1.52 Ac |
|  |  | RB | 27.0±0.55 Ba | 22.8±2.78 Aa | 25.3±1.18 Aa |
|  |  | B | 30.4±0.75 Ba | 30.9±1.17 Aab | 21.9±1.52 Bb |
|  | HN | R | 28.1±1.52 Aa | 24.0±2.17 Aab | 18.3±0.88 Ab |
|  |  | RB | 18.7±0.50 Bb | 20.2±1.15 Aa | 17.6±0.55 Ab |
|  |  | B | 17.8±2.55 Bab | 22.6±2.05 Aa | 15.2±0.20 Bb |

Table S2 **Concentration of nitrate and soluble sugars in lettuce root in response of duration of light exposure, nitrogen input and light quality.** Means ± standard deviation; different letters indicate differences at p = 0.05 according to Fischer’s l.s.d. with lower-case letters for comparison among exposure hour under same light quality, and capital letters for comparison among light treatments under same nitrogen input.

| Trait | Nitrogen | Light | Light exposure (h) | | |
| --- | --- | --- | --- | --- | --- |
|  |  |  | 0 | 6 | 14 |
| Nitrate concentration  (μg g^-1^ FW) | LN | R | 39.2±12.1 Ba | 52.9±14.5 ABa | 42.4±2.59 Ba |
|  |  | RB | 33.6±6.99 Bb | 40.5±5.19 Bb | 83.8±7.20 Aa |
|  |  | B | 84.0±3.45 Aa | 67.7±4.58 Ab | 77.4±5.92 Aab |
|  | HN | R | 366±21.6 Ba | 383±15.8 Ba | 378±23.6 Ba |
|  |  | RB | 367±38.5 Bb | 371±24.1 Bb | 452±26.0 Aa |
|  |  | B | 531±8.50 Aa | 457±6.67 Ab | 449±4.81 Ab |
| Soluble sugar concentration  (mg g^-1^ FW) | LN | R | 11.9±0.75 Bb | 20.2±0.93 Aa | 11.5±0.62 Bb |
|  |  | RB | 13.5±0.57 Bb | 14.2±0.83 Ba | 13.6±1.45 ABb |
|  |  | B | 19.0±0.71 Aa | 15.7±1.01 Bb | 15.6±0.42 Ab |
|  | HN | R | 9.76±0.63 Ab | 15.1±0.76 Aa | 9.55±0.86 Ab |
|  |  | RB | 8.49±0.61 Aa | 8.83±0.57 Ba | 9.32±0.30 Aa |
|  |  | B | 8.06±0.98 Ab | 9.98±1.15 Ba | 10.4±0.79 Aa |
